# Seasonal Role Reversal of Baer's Pochard in Waterbird Association Networks

**DOI:** 10.1002/ece3.74021

**Published:** 2026-08-04

**Authors:** Senhao Li, Fangzhong Lv, Heng Wu, Jianhua Hou

**Affiliations:** ^1^ College of Life Science Hebei University Baoding China; ^2^ Engineering Research Center of Ecological Safety and Conservation in Beijing‐Tianjin‐Hebei (Xiong'an New Area) of MOE Baoding China; ^3^ Hebei Basic Science Center for Biotic Interactions Baoding China

**Keywords:** Baer's Pochard, dynamic change, ecological network analysis, interspecific association, waterbird community

## Abstract

Interspecific associations are fundamental to understanding community structure and stability, holding critical implications for the conservation of endangered species. However, research on the network position of species within waterbird communities remains limited, particularly regarding the Critically Endangered Baer's Pochard ( *Aythya baeri* ). To address this gap, we conducted a systematic survey of the Baiyangdian Wetland (BYD) from May 2023 to March 2024, recording Baer's Pochard and 48 sympatric waterbird species. We constructed and analyzed interspecific association networks for both breeding and wintering periods using the Phi coefficient (*r*_*Φ*_) and Lambda coefficient (*L*_*B*_), and calculated the species association diversity index (*H*′) and community contribution rate (*C*_*i*_) to examine the network position of Baer's Pochard and its seasonal variation. Results indicated that, within the focal study year, the waterbird community in Baiyangdian exhibited a significant symmetrical positive association pattern in both seasons, consistent with community assembly influenced by environmental filtering, although additional ecological processes may also contribute. However, the network position of Baer's Pochard changed markedly between seasons: during the breeding season, it functioned as a core species, forming close associations with six other waterbird species and exhibiting higher *H*′ and *C*_*i*_ values, occupying a more central position in the network; during the wintering season, it shifted to a peripheral position, with reduced connectivity and relative network centrality, while the Eurasian Coot ( *Fulica atra* ) replaced it as the central node of the wintering network. Concurrently, the number and identity of core species varied dynamically across seasons, with some species maintaining stable core status and others undergoing positional shifts. These findings suggest that the structural position of endangered species within co‐occurrence networks may vary seasonally, although the interannual stability of this pattern requires further verification through long‐term monitoring. This study emphasizes that conservation strategies should account for both breeding and wintering periods, maintaining overall community stability while safeguarding critical microhabitats. The study provides a scientific basis for the targeted conservation of Baer's Pochard and offers a case reference for understanding the seasonal dynamics of network position of endangered waterbirds within communities.

## Graphical abstract

Seasonal Network Position Shift: Baer's Pochard exhibits a marked seasonal transition in its network position within the waterbird community—functioning as a core species with high connectivity during the breeding season, but shifting to a peripheral position with reduced network centrality during winter. Environmental Filtering Drives Community Assembly: The waterbird community in Baiyangdian shows significant positive associations in both seasons, consistent with shared habitat responses under environmental filtering, though the identity and number of core species change dynamically across seasons. Conservation implications: Conservation strategies should adopt a season‐specific approach—maintaining diverse breeding habitats and monitoring co‐occurring species during the breeding season, while identifying and protecting specialized microhabitats (deep‐water, ice‐free areas) critical for Baer's Pochard survival during winter.

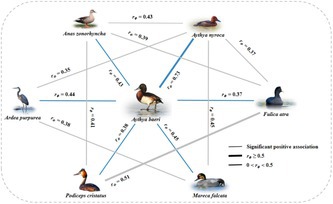

## 1 Introduction

Currently, from the perspective of conservation biogeography, the focus of conservation is gradually shifting from species‐centered approaches to the coordinated protection of overall ecosystem functions (Li et al. 2020). The concept of single‐species conservation is increasingly questioned, as overly targeted protection may inadvertently compromise the populations of sympatric species (Huang et al. 2024). Understanding species association networks is therefore critical for balancing single‐species conservation with ecosystem‐level stability (Bascompte and Jordano 2007). Environmental filtering, the process by which abiotic conditions select for species with similar environmental tolerances, has been shown to shape community assembly across diverse ecosystems (HilleRisLambers et al. 2012). In waterbird communities, habitat heterogeneity drives spatial co‐occurrence patterns (Elmberg et al. 1997). In contrast, identifying and maintaining core structural species within community networks is considered beneficial for enhancing the stability and disturbance resistance of the entire community (Bascompte and Jordano 2007; Fortuna et al. 2010). Therefore, identifying and protecting species spatial association networks is regarded as an effective pathway to coordinate conservation goals at both the species and ecosystem levels (Tylianakis et al. 2010; Wang, Que, et al. 2023). It should be emphasized, however, that association networks constructed from spatial co‐occurrence data cannot directly demonstrate mechanisms of interaction such as competition, predation, or regulation.

Interspecific association analysis is a core component of community ecology research, revealing how environmental filtering and biotic interactions jointly shape community structure (Wiegand et al. 2007; Kraft et al. 2015). Studying these relationships allows for a deeper understanding of population dynamics, community stability, and coexistence mechanisms (Wu et al. 2019; Liu et al. 2022). Positive spatial associations between species may stem from mutualism or social aggregation, but often reflect shared habitat preferences driven by environmental filtering (Wisz et al. 2013). Such shared preferences suggest potential niche overlap and competitive interactions between co‐occurring species (Calatayud et al. 2019). In waterbird communities, environmental filtering often drives co‐occurrence patterns, as species aggregate in high‐quality habitats offering optimal foraging and roosting conditions (Elmberg et al. 1997; Pöysä 1983). Consequently, studying interspecific spatial association networks is not only key to understanding community structure (Wiegand et al. 2007) but also aids in identifying resource utilization patterns and coexistence mechanisms among species, providing effective support for species conservation (Zhu et al. 2011).

However, although community network structures possess temporal dynamics (CaraDonna et al. 2017; Dupont et al. 2009), current association network studies mostly focus on static descriptions. There is less attention paid to how the core‐periphery roles of species within community networks undergo functional transitions across seasons. This knowledge gap is particularly critical given that waterbirds experience distinct ecological pressures across seasons. Interspecific association network analysis is increasingly applied in diverse taxa (Yuan and Wang 2023; Huang et al. 2024), yet research on waterbird communities remains relatively limited (Shi 2015), especially regarding seasonal dynamics in the network roles of endangered species. While some studies have explored the effects of residency type and season on avian interspecific associations (Dong et al. 2013), these studies largely fail to focus on endangered waterbird species or quantify seasonal shifts in network centrality. In particular, there is a lack of in‐depth analysis regarding the directionality of interspecific associations, making it difficult to determine whether an endangered species functions as a community dependent or a core maintainer, and precluding a systematic understanding of the seasonal dynamics of its role within community networks. Waterbirds face distinctly different selection pressures during breeding and wintering seasons. During breeding, nest site competition and intraspecific territoriality are primary drivers (Pöysä 1983), whereas resource scarcity and thermal stress dominate in winter (Beauchamp 2014; Fox and Madsen 2017). Investigating the dynamic changes in the network position of critically endangered waterbirds across seasons is therefore a scientific problem that urgently needs to be addressed.

The focal species of this study, Baer's Pochard ( *Aythya baeri* ), belongs to the family Anatidae of the order Anseriformes and is listed as Critically Endangered (CR) on the IUCN Red List (IUCN 2025). Current research on Baer's Pochard concentrates on population monitoring (Wang et al. 2012; Li et al. 2022), breeding and wintering behaviors (Wei et al. 2020), and distribution surveys and habitat suitability assessments (Jiang et al. 2021; Tian et al. 2024). There is a severe lack of understanding regarding its ecological associations within sympatric waterbird communities, which affects the effective implementation of conservation efforts. This study focuses on Baiyangdian Wetland (BYD), a habitat for Baer's Pochard located in the Xiong'an New Area, Hebei Province. It is the largest freshwater wetland in the North China Plain and a key node on the East Asian‐Australasian Flyway (Gu et al. 2025), serving as a vital habitat for Baer's Pochard and numerous other waterbirds. However, systematic research on the waterbird community structure in this region remains insufficient, particularly lacking analysis of ecosystem function from the perspective of interspecific association networks. This has led to a lack of sufficient scientific basis for conservation management strategies. Therefore, based on systematic field surveys conducted from May 2023 to March 2024, this study constructed interspecific association networks for the breeding and wintering seasons to address the following questions: (1) What relative network position does Baer's Pochard occupy in the waterbird co‐occurrence network of BYD during the breeding and wintering seasons, respectively? (2) Are these seasonal differences consistent with the overall co‐occurrence patterns and shared habitat responses of the community? (3) Based on the above results, how can more targeted conservation recommendations be provided for Baer's Pochard from the perspective of season‐specific habitat management? This study aims to provide scientific support for the targeted conservation of Baer's Pochard and to supplement our understanding of its seasonal shifts in community network position from a co‐occurrence perspective.

## 2 Methods

### 2.1 Study Area

**FIGURE 1:**
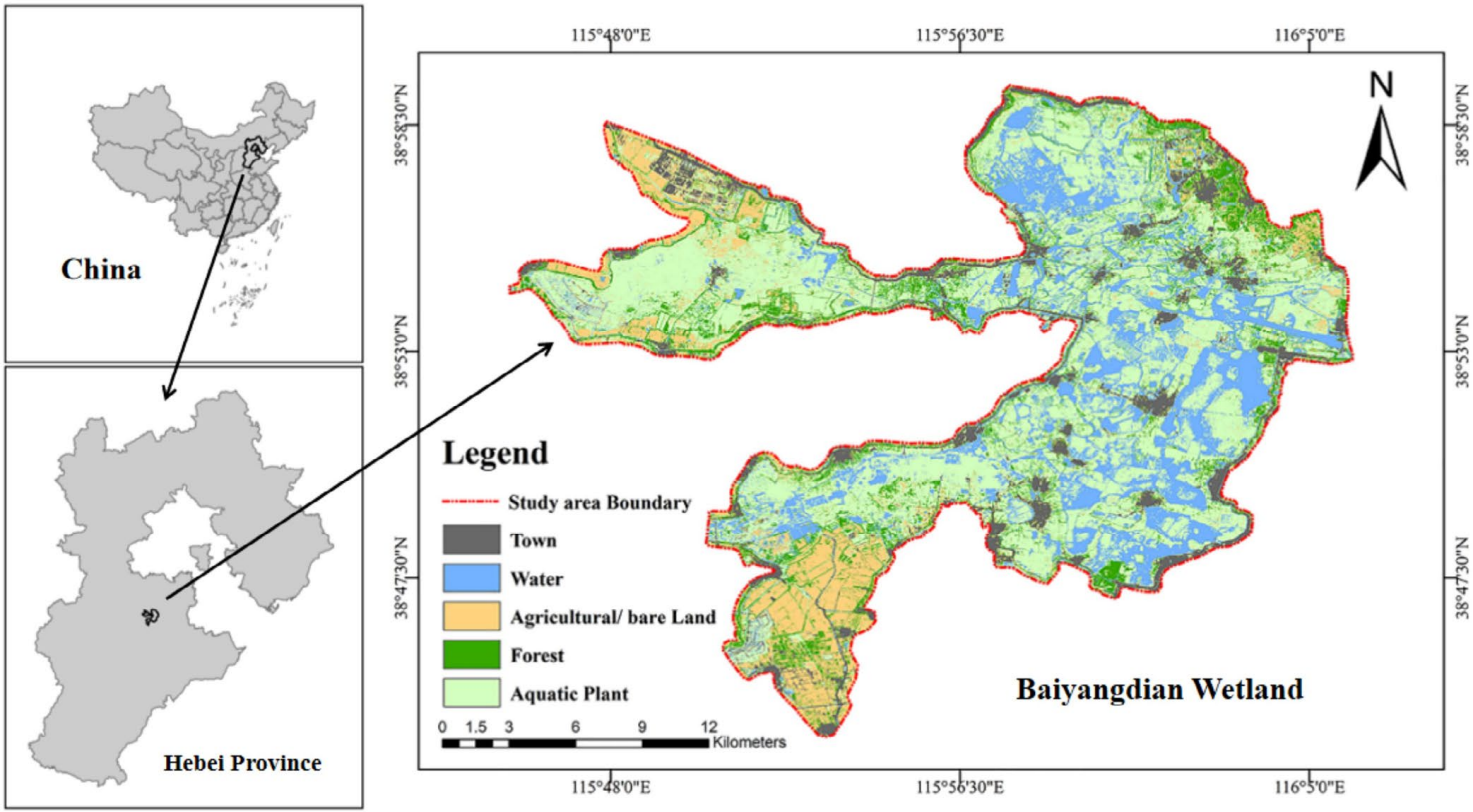
Location of Baiyangdian Wetland in China.

BYD (38°43′ N–38°49′ N, 115°45′ E–116°07′ E) is located within the Xiong'an New Area in the north‐central part of the North China Plain (Figure 1) and is the largest freshwater lake wetland in North China (Yang et al. 2020). BYD belongs to a warm temperate semi‐humid continental monsoon climate with distinct seasons: dry and windy in spring, hot and rainy in summer, crisp in autumn, and cold and dry in winter (Wang, Que, et al. 2023). The wetland consists of 143 lakes of varying sizes and over 3700 ditches. The terrain is generally flat, tilting slightly from northwest to southeast (Zheng et al. 2017). As a typical shallow macrophytic lake, BYD possesses abundant aquatic vegetation, fish, and benthic resources, providing stable food sources and habitats for birds.

### 2.2 Field Investigation and Data Collection

In this study, waterbirds are defined as wetland bird species that primarily depend on wetland aquatic habitats—including open water, shallow water zones, and emergent vegetation belts—for foraging and roosting (Wetlands International 2018). The waterbirds included in the analysis comprised Baer's Pochard and 48 sympatric species recorded during field surveys in the Baiyangdian Wetland, belonging to 6 orders and 10 families: Anseriformes, Podicipediformes, Gruiformes, Pelecaniformes, Suliformes, and Charadriiformes. All species included are listed in Table S1.

**FIGURE 2:**
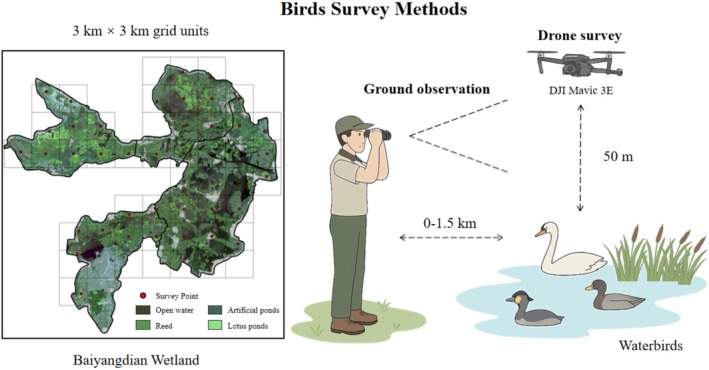
Distribution of survey points and schematic of survey methods. Left: Schematic of the 3 km × 3 km grid units in Baiyangdian Wetland; Right: Illustration of ground observation and drone‐assisted survey methods.

The Baiyangdian Wetland was divided into 46 grid units of 3 km × 3 km, with the center point of each grid serving as a fixed survey site; the minimum distance between adjacent sites was 3 km (Figure 2). Species occurrence was recorded using site × survey round as the sampling unit. Surveys were conducted using a combination of ground observation and drone‐assisted methods. During the breeding season (May–August 2023) and the wintering season (December 2023–March 2024), four repeated survey rounds (one per month) were conducted at all 46 sites per season, yielding a total of 8 rounds and 368 individual surveys across both seasons. Each survey was carried out during two time periods, 06:00–09:00 and 16:00–19:00, with each fixed‐point observation lasting 30 min. Survey timing was adjusted by ±1 h across seasons according to sunrise and sunset times, while maintaining two consistent time windows and a uniform observation duration per visit. Each survey round was preferentially conducted under clear, calm weather conditions. Within each season, all sites were surveyed synchronously to minimize temporal drift bias. The same personnel and team size were maintained across all survey rounds.

In areas with open views, surveyors used 12 × 50 binoculars for fixed‐point observation. For blind spots difficult for personnel to reach, such as deep reed marshes, a DJI Mavic 3E drone was used to hover and observe at a height of 50 m above the survey site; this altitude has been demonstrated to cause minimal disturbance to birds (Barr et al. 2020; Irigoin‐Lovera et al. 2019). Ground and drone observation results were de‐duplicated and merged within the same time window to generate the species record for each site per survey round. Groups potentially moving between sites were cross‐checked using time stamps and GPS coordinates, and were assigned to the site of first detection when necessary to avoid double‐counting. This integrated ground–air survey approach effectively covered typical habitats within a radius of 0–1.5 km, including open lake areas, artificial ponds, reed platforms, and lotus ponds, ensuring comprehensive data coverage.

### 2.3 Interspecific Association Calculation and Multiple Testing Correction

The biological integrity of a region reflects the completeness of the ecosystem and is the basis for the community's existence. Therefore, the Variance Ratio (*V*_R_) was first used to analyze overall integrity, with the calculation process referring to Schluter (1984). To further parse specific spatial relationships between species, the Phi coefficient (*r*_*Φ*_) was used to measure the strength of interspecific association (Siegel and Castellan Jr 1988). This coefficient is calculated based on a contingency table of binary data (0/1), with significance determined via the Chi‐square test (*χ* ^2^). In this study, a total of 43 waterbird species were recorded during the breeding season, yielding 903 species pairs; 27 waterbird species were recorded during the wintering season, yielding 351 species pairs. Pairwise association tests were conducted separately for each season. Only positive associations with statistical significance (*r*_*Φ*_ > 0, *p* < 0.05) were retained for subsequent network construction (Wang, Shen, et al. 2023).

Prior to calculating the Phi coefficient, the following values were first computed for each species pair: *a*, the number of sites where both species co‐occurred; *b*, the number of sites where only the first species occurred; *c*, the number of sites where only the second species occurred; and *d*, the number of sites where neither species occurred. The formula for the Phi coefficient is as follows:

$$r_{\Phi }=\frac{\mathrm{ad}-\mathrm{bc}}{\sqrt{\left(a+b\right)\left(b+c\right)\left(a+c\right)\left(b+d\right)}}$$

where *r* Φ is the Phi coefficient, used to measure the strength of association between species. The value of *r* Φ ranges from [−1, 1], with larger values indicating stronger associations. Significance testing of the coefficient was subsequently conducted using the following calculation:

$$\chi^{2}={\frac{N\left(|\mathrm{ad}-\mathrm{bc}|\frac{N}{2}\right)}{\left(a+b\right)\left(b+c\right)\left(a+c\right)\left(b+d\right)}}^{2}$$

where *χ* ^2^ is the Chi‐square value, and *N* is the total number of survey sites, *N* = *a* + *b* + *c* + *d*. When *χ* ^2^ < 3.841, *p* > 0.05, and the *r* Φ value is not statistically significant. When *χ* ^2^ ≥ 3.841, *p* ≤ 0.05, and the *r* Φ value is statistically significant.

Given that simultaneously testing a large number of species pairs may lead to false positive inflation, the Benjamini‐Hochberg method (BH) (Benjamini and Hochberg 1995) was applied to correct the *p* values of all pairwise Chi‐square tests for the false discovery rate. Only positive association pairs with a corrected *q* < 0.05 and *r* Φ > 0 were retained for subsequent network construction.

### 2.4 Analysis of Pairwise Spatial Association Attributes and Construction of Spatial Association Networks

Given that the Phi coefficient only reflects symmetrical associations, this study introduced the Lambda coefficient (*L*_*B*_) to further detect asymmetry in associations, that is, determining whether the presence of species A unidirectionally predicts the presence of species B (Li, Li, and Liu 2024). By calculating the predictive upper limit (*L*_*B*_) and lower limit (*λ*_*B0*_) and conducting significance tests, we identified whether dependency or unidirectional benefit relationships exist within the community. If no significant asymmetry was detected, a neutral interpretation was maintained, and no strong inferences were drawn regarding directional influences between species.

Following BH correction, no significant negative association pairs were detected in either season (*q* ≥ 0.05); consequently, the association networks were composed exclusively of significant positive association pairs. Network nodes comprised all species recorded in a given season; species that did not form associations with any other species were recorded as isolated nodes. However, given the large number of nodes and edges in the full network, direct visualization would compromise figure readability. Therefore, the results figures present only the sub‐network centered on Baer's Pochard, displaying species with significant associations with Baer's Pochard and the association relationships among those species; species not associated with Baer's Pochard and their inter‐associations are not shown. Based on significant positive association data, NetDraw software (Borgatti 2002) was used to plot the species spatial association network.

### 2.5 Calculation of Species Association Diversity and Community Contribution Rate

To quantify the status of each species in the network, the species Association Diversity Index (*H*′) and Community Contribution Rate (*C*_*i*_) were calculated (Wang, Shen, et al. 2023). Here, *H*′ reflects the richness and evenness of a species' associated partners, while *C*_*i*_ represents the relative contribution of that species to the information content of the entire network. Following the method of Huang et al. (2024), species with an *H*′ value higher than the network average were defined as Core Species, while those below were defined as Peripheral Species. To further test the sensitivity of core species classification to threshold selection, we additionally applied two alternative criteria for repeated identification of core species: *H*′ above the network mean + 0.5 SD, and *H*′ above the network median. We then compared whether the network role of Baer's Pochard remained consistent across different classification criteria, thereby evaluating the robustness of its seasonal positional shift.

It should be noted that *H*′ and *C*_*i*_ reflect the structural position and relative connectivity characteristics of species within the co‐occurrence network, and their values may be influenced by species occurrence frequency and spatial distribution range. Therefore, the term “core species” in this study refers to core nodes in the structural sense of the network, and does not directly equate to keystone species in the ecological sense.

The formula for calculating the species Association Diversity Index (*H*′) is as follows:

$$H^{'}=-\sum_{i=1}^{S}\text{PilnPi}$$

where $H^{'}$represents the Shannon–Wiener index, *S* is the number of direct positive associations generated by a given species, and *Pi* is the ratio of the association coefficient of species pair *i* to the sum of all association coefficients of that species.

The formula for the Community Contribution Rate (*C*_*i*_) is as follows:

$$C_{i}=\frac{H_{i}^{'}}{\sum_{i=1}^{S}H_{i}^{'}}\times 100\%$$

where *S* is the number of species, and $H_{i}^{\prime }$ is the Shannon–Wiener index of species *i*.

All statistical analyses were completed in R 4.4.3 (R Core Team 2025). We used the dplyr package (Wickham et al. 2023) for data cleaning and organization, and the spaa (Zhang 2016) and psych (Revelle 2025) packages to calculate interspecific association indices and conduct statistical tests. Data visualization was completed using the ggplot2 package (Wickham 2016).

## 3 Results

### 3.1 Overview of the Baiyangdian Waterbird Community Survey

This study recorded a total of 48 waterbird species, including Baer's Pochard and sympatric species, belonging to 6 orders and 10 families in BYD between May 2023 and March 2024. Of these, 43 species were recorded during the breeding season and 27 during the wintering season. Classified by residency type, the species included 12 residents, 13 summer visitors, 9 winter visitors, and 14 passage migrants (Table S1). The breeding season community was predominantly composed of species from the families Anatidae, Ardeidae, and Podicipedidae, while the wintering season community was dominated by Anatidae species along with some Ardeidae and Rallidae species. Baer's Pochard occurred at 20 survey sites during the breeding season and at 16 sites during the wintering season, indicating seasonal differences in its distributional range. Waterbird records were primarily concentrated in habitat types such as open water, artificial ponds, reed platforms, and lotus ponds. Overall, the diverse habitat types and their landscape mosaic patterns within BYD provide an important context for understanding the seasonal differences in the subsequent waterbird co‐occurrence networks.

### 3.2 Overall Association of the Bird Community

Variance Ratio analysis indicated that the waterbird community in BYD exhibited a significant non‐random positive association pattern in both the breeding and wintering seasons. The *V*_R_ values for both seasons were significantly > 1 (Breeding: *V*_R_ = 1.87, *W* = 155.2, *p* < 0.01; Wintering: *V*_R_ = 2.15, *W* = 189.8, *p* < 0.01). This result reveals that the waterbird community in the study area is a structured entity, with species co‐occurrence frequencies significantly higher than random expectations.

### 3.3 Interspecific Association Network in the Breeding Season

In the breeding season, 43 waterbird species were recorded, forming 52 statistically significant positive associations after BH correction (*χ* ^*2*^ ≥ 3.841, *q* < 0.05) (Table S2). All significant associations were symmetrical positive associations, indicating that species tended to co‐occur spatially, more likely reflecting shared responses to similar habitat conditions or overlapping habitat utilization; the co‐occurrence results of this study do not constitute evidence of direct ecological interactions such as competition or predation.

**FIGURE 3:**
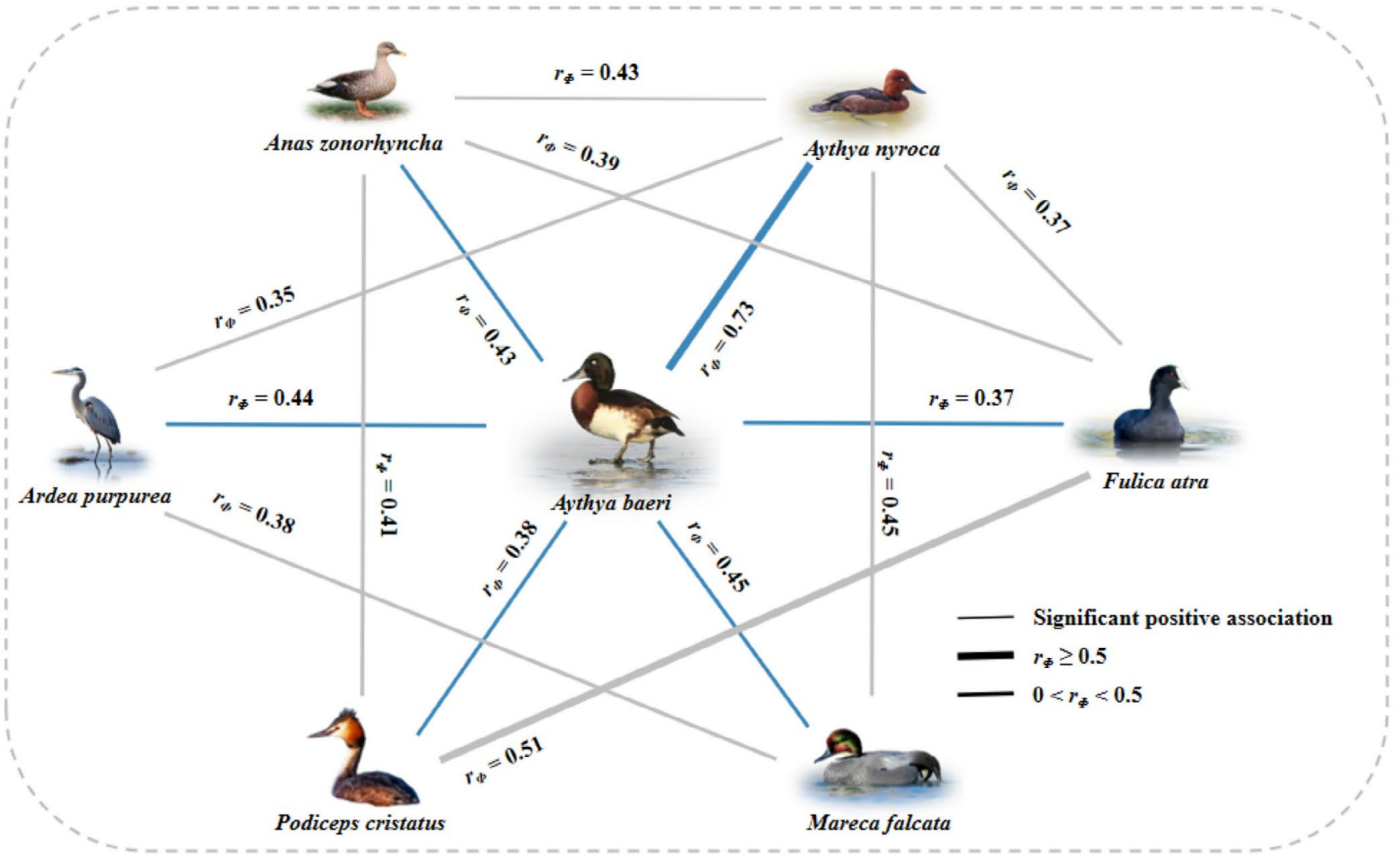
Interspecific association network of Baer's Pochard during the breeding season at Baiyangdian. Nodes represent species exhibiting significant positive associations with Baer's Pochard during the breeding season. Lines between nodes indicate significant positive associations between species. Blue lines represent direct associations with Baer's Pochard. Gray lines represent associations among other species in the network. Thick lines indicate *r*_*Φ*_ ≥ 0.5, representing stronger positive associations. Thin lines indicate 0 < *r*_*Φ*_ < 0.5, representing weaker positive associations.

In this network, Baer's Pochard exhibited characteristics of a core species, with its species association diversity index (*H*′ = 1.76) higher than the network average (1.11). The Phi coefficient results showed significant positive associations with 6 species. The strength of association, from high to low, was: Ferruginous Duck ( *Aythya nyroca* ) (*r*_*Φ*_ = 0.73, *q* < 0.05), Falcated Duck (*Mareca falcata*) (*r*_*Φ*_ = 0.45, *q* < 0.05), Purple Heron ( *Ardea purpurea* ) (*r*_*Φ*_ = 0.44, *q* < 0.05), Eastern Spot‐billed Duck ( *Anas zonorhyncha* ) (*r*_*Φ*_ = 0.43, *q* < 0.05), Great Crested Grebe ( *Podiceps cristatus* ) (*r*_*Φ*_ = 0.38, *q* < 0.05), and Eurasian Coot ( *Fulica atra* ) (*r*_*Φ*_ = 0.37, *q* < 0.05) (Figure 3). Together, these formed the spatial association species group of Baer's Pochard. The number of associated species for Baer's Pochard ranked fifth among all 43 species, highlighting its important role in community connectivity during the breeding season.

The entire breeding season network identified 14 core species (*H*' > 1.11), with a cumulative contribution rate to the community as high as 84.42% (Table 1). Baer's Pochard was among these core species, with an association diversity index (*H*') of 1.76 and six direct associations with other waterbird species, indicating that it occupied a core structural position within the breeding‐season co‐occurrence network. Eight waterbird species had both association diversity indices and community contribution rates of zero (*H*′ = 0, *C*_*i*_ = 0) (Table 1), indicating they are at the community periphery with a weak role in maintaining network structure. The six waterbird species associated with Baer's Pochard also individually formed extensive positive associations with various other waterbird species, suggesting that while Baer's Pochard has direct spatial associations with these six species, it also maintains indirect associations with species in their respective groups through them. Lambda asymmetric association test results showed that all species pair associations were symmetrical positive associations (Table S2), with no significant unidirectional dependency found (*p* > 0.05), indicating no predictive relationship between them.

**TABLE 1:** Association diversity and community contribution rates of core species in the breeding season.

Number	Species	Associated species	Shannon–Wiener index (*H*′)	Contribution (*C*_*i*_) (%)
1	*Aythya nyroca*	11	2.37	7.90
2	*Anas zonorhyncha*	8	2.07	6.90
3	*Mareca strepera*	8	2.07	6.89
4	*Ardea purpurea*	7	1.94	6.47
5	*Gallinula chloropus*	7	1.94	6.45
6	*Anas platyrhynchos*	7	1.94	6.45
7	*Podiceps cristatus*	6	1.79	5.95
8	*Ardea cinerea*	6	1.78	5.94
9	*Mareca falcata*	6	1.78	5.94
10	*Egretta garzetta*	6	1.77	5.91
11	*Aythya baeri*	6	1.76	5.87
12	*Tachybaptus ruficollis*	4	1.38	4.59
13	*Fulica atra*	4	1.38	4.59
14	*Ardeola bacchus*	4	1.37	4.57

### 3.4 Interspecific Association Network in the Wintering Season

Entering the wintering season, the species composition and network structure of the BYD waterbird community changed significantly, with the number of recorded waterbird species dropping to 27. The community overall still exhibited positive associations (*r* Φ> 0), but significant positive associations decreased to 31 (*q* < 0.05) (Table S3).

**FIGURE 4:**
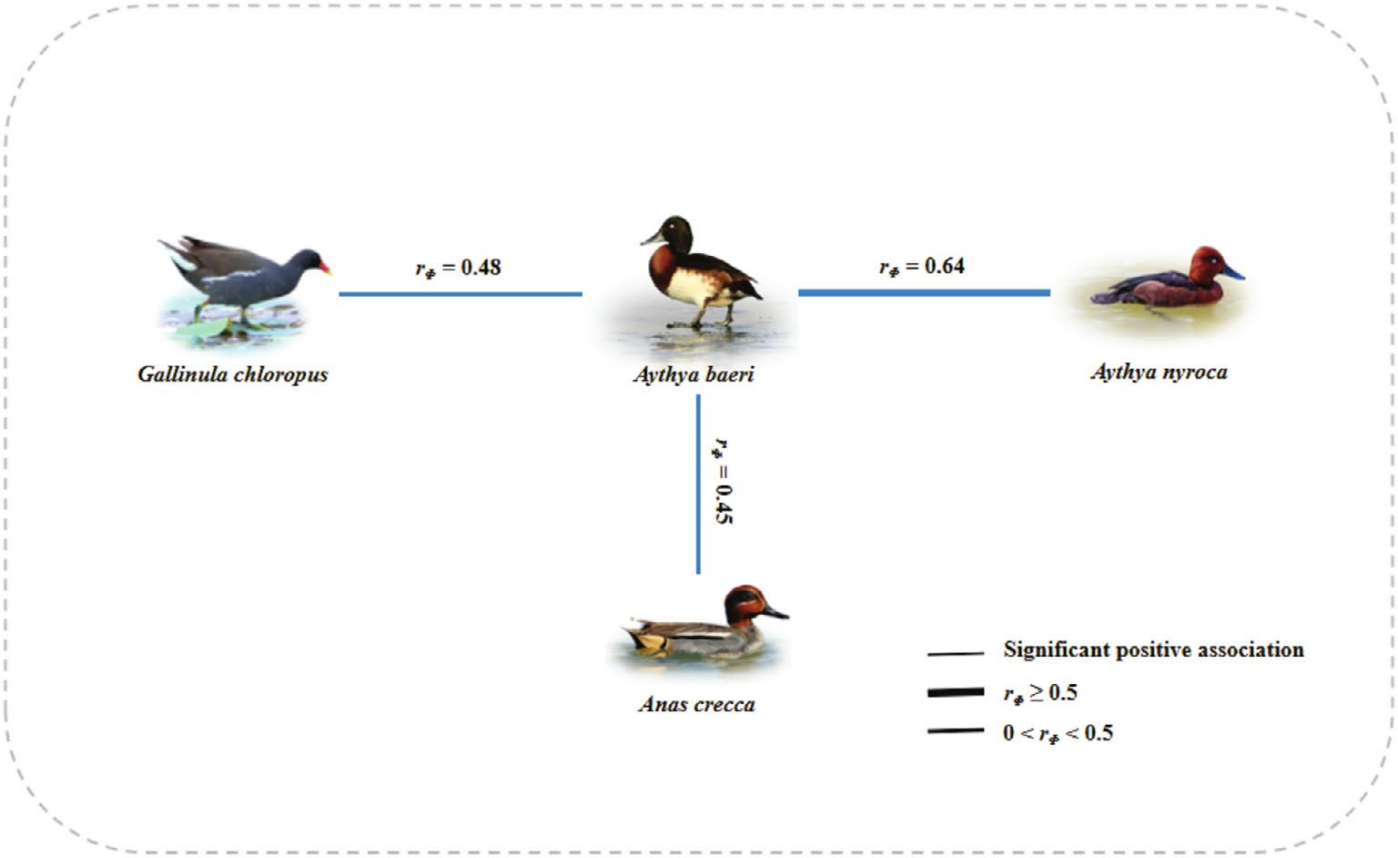
Interspecific association network of Baer's Pochard during the wintering season at Baiyangdian. Nodes represent species exhibiting significant positive associations with Baer's Pochard during the wintering season. Lines between nodes indicate significant positive associations between species. Blue lines represent direct associations with Baer's Pochard. Thick lines indicate *r*_*Φ*_ ≥ 0.5, representing stronger positive associations. Thin lines indicate 0 < *r*_*Φ*_ < 0.5, representing weaker positive associations.

In sharp contrast to the breeding season, Baer's Pochard transformed from a core species to a peripheral species in the wintering network. Its species association diversity index was lower than the season's network average (*H*′ < 1.56), showing significant positive associations with only 3 species: Ferruginous Duck (*r*_*Φ*_ = 0.64, *q* < 0.05), Common Moorhen ( *Gallinula chloropus* ) (*r*_*Φ*_ = 0.48, *q* < 0.05), and Eurasian Teal ( *Anas crecca* ) (*r*_*Φ*_ = 0.45, *q* < 0.05) (Figure 4). This result clearly shows that the influence of Baer's Pochard on community structure significantly weakened in winter. The number of core species in the wintering network decreased to 7 (Table 2). Notably, the Eurasian Coot possessed the highest number of associated species (11 species) and community contribution rate (*H*′ = 2.40, *C*_*i*_ = 17.10) in this season (Table 2, Figure 4), replacing the roles of multiple breeding season core species and becoming the most critical structural species in the wintering community.

**TABLE 2:** Association diversity and community contribution rates of core species in the wintering season.

Number	Species	Associated species	Shannon–Wiener index (*H*′)	Contribution (*C*_*i*_) (%)
1	*Fulica atra*	11	2.40	17.10
2	*Egretta garzetta*	6	1.64	11.72
3	*Spatula querquedula*	6	1.64	11.72
4	*Botaurus stellaris*	6	1.64	11.72
5	*Sibirionetta formosa*	6	1.64	11.72
6	*Spatula clypeata*	6	1.64	11.72
7	*Anas acuta*	6	1.64	11.72

### 3.5 Association Symmetry Analysis Lambda

Lambda coefficient tests showed that among all significant positive association species pairs in both breeding and wintering seasons, no significant asymmetric relationships were found (*p* > 0.05) (Tables S2 and S3). This result indicates that the spatial co‐occurrence relationships between Baer's Pochard and other waterbird species primarily manifest as symmetrical interactions or are driven by shared environmental requirements, without exhibiting obvious unidirectional dependence or predictive relationships.

### 3.6 Sensitivity Analysis Results

Sensitivity analysis revealed that under different threshold criteria, Baer's Pochard generally maintained a core position in the breeding season network while remaining marginalized in the wintering season, demonstrating that its seasonal network position differences are not entirely contingent upon a single threshold setting (Table 3).

**TABLE 3:** Sensitivity analysis of the seasonal network position of Baer's Pochard under different threshold criteria.

Season	*H*′ of Baer's Pochard	Network mean	Network median	Network mean + 0.5 SD
Breeding	1.76	1.11	1.37	1.53
Wintering	0.48	0.52	0	0.92

## 4 Discussion

Based on a survey covering one complete annual cycle, this study found that the structural position of Baer's Pochard within the BYD waterbird co‐occurrence network exhibited marked differences between the breeding and wintering seasons. Our results showed that Baer's Pochard occupied a more core network position during the breeding season, while shifting to a peripheral position during the wintering season. This result suggests that assessments of the community role of endangered species should not be based solely on a single season, emphasizing that the dynamic nature of a species' position in the community network must be considered when evaluating the conservation value of endangered species.

### 4.1 Core Status of Baer's Pochard in Breeding Season and Potential Resource Overlap

Our results show that Baer's Pochard becomes a core species in the BYD waterbird community during the breeding season, showing significant symmetrical positive associations with various taxa (Figure 3). This finding indicates that Baer's Pochard is not only an important node in the community structure during the breeding season but may also function as a structural species (Power et al. 1996) through mechanisms such as complementary resource use, shared predation risk, and habitat sharing. However, this mechanism requires further validation combined with behavioral observations, dietary, and resource utilization data. This pattern of species coexistence driven by environmental filtering is consistent with broader patterns in bird community assembly. Environmental filtering dominates community assembly in resource‐limited or stressful environments, whereas biotic interactions become more influential in productive habitats (Kraft et al. 2015). In waterbird communities specifically, habitat heterogeneity and seasonal resource availability are primary drivers of co‐occurrence patterns (Pöysä 1983; Elmberg et al. 1997), with network structure dynamically adjusting in response to environmental gradients and seasonal shifts (Liang et al. 2021). These findings underscore the importance of environmental filtering as a key mechanism shaping waterbird community networks, although the relative contribution of competition or facilitation in structuring local assemblages requires further investigation through experimental or behavioral studies. Secondly, unlike the conclusion proposed by Tylianakis et al. (2010) that “endangered species are mostly peripheral members of the community,” Baer's Pochard is a core species in the breeding season. This difference may stem from the ecological restoration of BYD in recent years, which has improved habitat quality (Gao et al. 2024; Liang et al. 2023), enabling Baer's Pochard to attain relatively higher network connectivity within the local community.

However, its core status is accompanied by potential ecological risks. Baer's Pochard showed the strongest spatial association with its congeneric relative, the Ferruginous Duck, with a Phi coefficient as high as 0.73 (Figure 3). Between closely related species with high niche overlap, this presages intense resource competition (Connell 1980; Pianka 1974), and the two pochard species exhibit certain niche overlaps in food resources and nest site selection (Cui 2024). Given the potential niche similarity between the two species, future research should strengthen synchronized monitoring of resource conditions and reproductive success of both species at key breeding sites during the breeding season to assess whether resource limitation or interference effects exist. Dhondt (2012) noted that when the population of closely related species in a bird community increases, they often reduce the reproductive success of the focal species through resource competition or interference behavior. This pattern of potential competition under strong association requires that conservation practice should extend beyond the superficial coexistence of species to address the underlying interspecific dynamics, avoiding the implicit suppression of the reproductive success of critically endangered species caused by the excessive expansion of dominant related species. Although this study infers potential competition based on spatial association, future verification combined with behavioral observations is needed.

### 4.2 Network Marginalization of Baer's Pochard in Wintering Season and Conservation Implications

In sharp contrast to the breeding season, Baer's Pochard shifted to a peripheral position in the wintering co‐occurrence network, accompanied by a marked decline in its community contribution rate. Such seasonal shifts are consistent with previous ecological network studies showing that network structure and the identity of structurally important species are inherently dynamic through time rather than static, as seasonal changes in species composition, resource availability, and habitat conditions reorganize interaction or co‐occurrence networks (Dupont et al. 2009; CaraDonna et al. 2017; Trøjelsgaard and Olesen 2016). Therefore, the shift in Baer's Pochard from a core to a peripheral position should be interpreted as part of seasonal community reorganization rather than an exceptional characteristic of this species. The reduced structural connectivity of Baer's Pochard within the wintering co‐occurrence network declined, possibly related to its enhanced preference for specific microhabitats. In addition, Baer's Pochard is a migratory species, and seasonal redistribution of individuals between breeding and wintering areas may also contribute to the reduced occurrence frequency observed within BYD during winter. In the present study, the number of occupied survey sites declined from 20 during the breeding season to 16 during the wintering season, suggesting that seasonal population redistribution may partially contribute to the observed reduction in network connectivity. This role shift aligns with predictions from niche theory: as resource availability declines in winter, habitat specialists often face greater survival pressure than generalists (Clavel et al. 2011). Compared to the Eurasian Coot, which gathers in large flocks, Baer's Pochard shows a stronger tendency for habitat specialization during the wintering period, preferring specific deep‐water, ice‐free ponds (Li, Ma, et al. 2024; Liu et al. 2020). This dependence on microhabitats limits the frequency of its spatial contact with other species, leading to the marginalization of its network status.

This seasonal role reversal provides a new perspective for conservation strategies for Baer's Pochard. In the breeding season, conservation efforts should focus on maintaining a healthy, species‐diverse wetland ecosystem, particularly on sustaining the quality and spatial connectivity of key breeding habitats, and conducting synchronized monitoring of species with high co‐occurrence with Baer's Pochard. Studies have shown that the expansion of dominant species in a community can lead to the decline of other species, thereby reducing overall diversity and ecosystem function (Mccarthy et al. 2006; Post and Forchhammer 2002; Prins and Van Der Jeugd 1993). However, in the wintering season, conservation strategies must be more precise, focusing on identifying and protecting “microhabitats” that meet its specific needs (Bosco et al. 2022), as these areas may not be fully covered by traditional protected area zoning based on total waterbird counts. This is similar to conclusions from conservation studies on other flagship species (e.g., Takin *Budorcas taxicolor* , Sambar *Rusa unicolor* ) (Li et al. 2022; Yang et al. 2021), namely that understanding the relationships of the target species within the community is a prerequisite for formulating effective management measures.

Traditional conservation strategies are often based on breeding habitat requirements while neglecting wintering community structure. However, studies show that community composition and diversity in the wintering period are also significantly influenced by habitat conditions and environmental changes (Hamza et al. 2024; Lehikoinen et al. 2021). This study suggests that although Baer's Pochard is no longer a core species in winter, its survival depends on the stability of the community as a whole. Therefore, conservation measures should account for both breeding and wintering periods, avoiding bias toward a single timeframe. Additionally, the migration of some migratory birds out of BYD in winter reduces the integrity of the community network. These factors may also underestimate the potential associations of Baer's Pochard in the wintering season.

### 4.3 Community Network Assembly and Species Turnover Driven by Environmental Filtering

This study shows that the BYD waterbird community exhibits significant positive association patterns (VR > 1) in both seasons, consistent with community assembly driven by environmental filtering (Kraft et al. 2015; Blanchet et al. 2020). Within BYD, spatial heterogeneity in water depth, vegetation cover, ice‐free areas, and food resources creates distinct habitat patches. These patches favor species with similar resource requirements, leading to non‐random co‐occurrence patterns (Wisz et al. 2013). The higher VR value in the wintering season (VR = 2.15) compared to the breeding season (VR = 1.87) may reflect more concentrated suitable habitats and intensified resource limitation during winter (Beauchamp 2014; Krause and Ruxton 2002). Field observations support this habitat‐filtering interpretation: Although Eurasian Coots tend to move in large flocks of dozens or even hundreds, ducks and grebes in small groups, and herons and shorebirds mostly solitarily or in small groups, we recorded up to 12 different species coexisting at a single survey point simultaneously. This multi‐species aggregation is more likely a shared response to high‐quality habitats rather than active social behavior between species (Fretwell and Lucas 1969; Elmberg et al. 1997; Pöysä 1983). The absence of significant asymmetric associations (*p* > 0.05; Appendix Tables S2, S3) further indicates that co‐occurrence primarily reflects environmental filtering rather than directional dependencies such as facilitation or obligate commensalism.

This study found that the number of core species in the BYD waterbird community changed significantly between breeding and wintering seasons, decreasing from 13 to 7 (Table 2). This aligns with existing research stating that community network structure is dynamic on a temporal scale, and the identity of core species may remain stable or shift across different periods (Olesen et al. 2007; Dupont et al. 2009; CaraDonna et al. 2017). In this study, the Little Egret and Northern Pintail maintained core status in both periods, possibly related to their wide niches and strong tolerance to resource fluctuations (Dupont et al. 2009; Tylianakis et al. 2010). Although the Little Egret ( *Egretta garzetta* ) and Northern Pintail ( *Anas acuta* ) are traditionally classified as summer visitors and passage migrants in the study area, respectively, surveys during the study period found both species inhabiting and foraging in BYD throughout the year; therefore, their residency status was designated as resident in this study (Table S1).

Conversely, the Ferruginous Duck's core status dropped to the lowest level in the wintering season, with its community contribution rate also markedly reduced. This may be related to its high dependence on specific food resources such as arthropods (Cui 2024); such resources decrease substantially in BYD during winter, leading to niche contraction. In contrast, dietary analysis shows that the primary food source of Baer's Pochard is fish (Cui 2024). Fish resources remain relatively abundant year‐round in BYD, allowing Baer's Pochard to maintain a certain level of community association despite its marginalized network position during winter. This pattern may indicate that Ferruginous Duck occupied a narrower range of suitable winter habitats or exhibited fewer opportunities for spatial co‐occurrence than Baer's Pochard during the study period. However, peripheral positions in co‐occurrence networks should not be directly interpreted as evidence of ecological specialization or greater vulnerability to global environmental change. Such interpretations require additional evidence on habitat specialization, resource use, and demographic responses (Clavel et al. 2011).

Meanwhile, species such as the Garganey (*Spatula querquedula*) and Northern Shoveler (*Spatula clypeata*) transformed from peripheral to core species in the wintering season, and the Eurasian Coot's core position rose to the highest (Tables 1 and 2). Taking the Eurasian Coot as an example, it was widely distributed across all survey points during winter and frequently formed large flocks. This high abundance and widespread distribution substantially increased its co‐occurrence probability with other species, thereby functioning as a more central node in the network (Martín González et al. 2010; Vázquez et al. 2009). This result demonstrates that the network roles of species are not static but undergo reorganization with changes in season, abundance, and distribution patterns (Blüthgen et al. 2008; Fortuna and Bascompte 2006; Trøjelsgaard and Olesen 2016). More generally, species abundance and occurrence frequency may influence co‐occurrence network metrics because abundant species have a higher probability of being detected together with other taxa. Therefore, although higher H' values indicate stronger structural connectivity within the network, they may partly reflect differences in abundance rather than ecological importance alone. This possibility should be considered when interpreting seasonal changes in network position.

BYD encompasses wetland types subject to considerable anthropogenic management, including artificial ponds and lotus ponds. Human activities may influence waterbird co‐occurrence patterns by altering local water depth, vegetation structure, food supply, and disturbance levels. Therefore, the seasonal differences observed in this study, in addition to being driven by natural seasonal processes, may also be partly shaped by anthropogenic habitat management. In addition, although standardized survey protocols and repeated field surveys were adopted throughout the study, differences in species detectability among habitat types and seasons cannot be completely excluded. Dense emergent vegetation, seasonal water‐level changes, and differences in flocking behavior may influence detection probabilities and consequently affect observed co‐occurrence patterns. Future studies incorporating occupancy models or detection‐corrected survey methods would help further evaluate this potential source of bias.

## 5 Conclusion

Based on a survey covering one complete annual cycle, this study demonstrates that the Critically Endangered Baer's Pochard exhibits a marked seasonal network position shift from core in the breeding season to peripheral in the wintering season within the BYD waterbird co‐occurrence network. This dynamic is likely associated with seasonal changes in life‐history strategies, habitat use, and resource availability: High connectivity in the breeding season may increase the potential for spatial overlap with ecologically similar species, while marginalization in the wintering season reflects its dependence on specific microhabitats and the replacement of its core status by widespread species. Accordingly, we propose a “seasonal differentiation” conservation framework: during the breeding period, priority should be given to maintaining the quality and spatial connectivity of key breeding habitats, while conducting monitoring of species with high co‐occurrence with Baer's Pochard; during the wintering period, efforts should focus on identifying and protecting key microhabitats such as deep‐water or ice‐free areas upon which it depends. At the same time, this study has several limitations. First, the conclusions are primarily based on species spatial co‐occurrence patterns; while such association analysis can reveal potential interactions, it cannot directly confirm the specific underlying ecological mechanisms, such as competition, facilitation, or shared habitat preferences. Second, the geographic scope is limited to BYD; the generalizability of the conclusions to other breeding or wintering grounds of Baer's Pochard remains to be verified. Additionally, network metrics constructed from spatial co‐occurrence may be influenced by species occurrence frequency and spatial distribution breadth; species with widespread or high‐frequency occurrence are more likely to attain higher numbers of associations and *H*′ values. Therefore, the core species identified in this study primarily reflect their structural position within the co‐occurrence network and should not be directly interpreted as keystone species with causal regulatory roles. Finally, the data come from only one complete annual cycle, failing to cover the potential impact of inter‐annual environmental fluctuations on the community network.

We suggest that future research should focus on three directions: (1) Employing behavioral observations, dietary analysis, and individual tracking to deeply investigate the specific mechanisms driving interspecific association patterns; (2) Expanding the research scope to multiple key nodes along the migration route of Baer's Pochard and incorporating inter‐annual data to test whether its seasonal network position differences exhibit inter‐annual stability and broader applicability; (3) Conducting long‐term positioned monitoring to reveal dynamic laws of community networks on longer time scales and their responses to climate change and anthropogenic disturbances.

## Author Contributions

**Senhao Li:** conceptualization (equal), investigation (equal), methodology (lead), writing – original draft (lead). **Fangzhong Lv:** investigation (equal), software (equal). **Heng Wu:** investigation (equal), software (equal). **Jianhua Hou:** resources (equal), supervision (equal), writing – review and editing (equal).

## Funding

This work was supported by the National Science and Technology Major Project, 2026ZD1217700 and the Collaborative Innovation Center for Baiyangdian Basin Ecological Protection and Beijing‐Tianjin‐Hebei Sustainable Development; National Key Wildlife, including *Aythya baeri*, Protection Projects in Baiyangdian, HBQH‐2023‐ZB‐006.

## Conflicts of Interest

The authors declare no conflicts of interest.

## Supporting information


**Table S1:** Checklist of waterbird species sympatric with Baer's Pochard in Baiyangdian Wetland.

**Table S2:** Breeding network edges original.

**Table S3:** Wintering network edges original.

**Data S1:** ece374021‐sup‐0004‐DataS1.xlsx.

**Data S2:** ece374021‐sup‐0005‐DataS2.xlsx.

## Data Availability

The datasets used and analyzed during the current study are available from the corresponding author.

## References

[ece374021-bib-0001] Barr, J. R. , M. C. Green , S. J. DeMaso , and T. B. Hardy . 2020. “Drone Surveys Do Not Increase Colony‐Wide Flight Behaviour at Waterbird Nesting Sites, but Sensitivity Varies Among Species.” Scientific Reports 10: 3781. 10.1038/s41598-020-60543-z. 32123223 PMC7052279

[ece374021-bib-0002] Bascompte, J. , and P. Jordano . 2007. “Plant‐Animal Mutualistic Networks: The Architecture of Biodiversity.” Annual Review of Ecology, Evolution, and Systematics 38: 567–593. 10.1146/annurev.ecolsys.38.091206.095818.

[ece374021-bib-0003] Beauchamp, G. 2014. Social Predation: How Group Living Benefits Predators and Prey. 1st ed. Elsevier/Academic Press.

[ece374021-bib-0004] Benjamini, Y. , and Y. Hochberg . 1995. “Controlling the False Discovery Rate: A Practical and Powerful Approach to Multiple Testing.” Journal of the Royal Statistical Society. Series B, Statistical Methodology 57: 289–300.

[ece374021-bib-0005] Blanchet, F. G. , K. Cazelles , and D. Gravel . 2020. “Co‐Occurrence Is Not Evidence of Ecological Interactions.” Ecology Letters 23: 1050–1063. 10.1111/ele.13525. 32429003

[ece374021-bib-0006] Blüthgen, N. , J. Fründ , D. P. Vázquez , and F. Menzel . 2008. “What do Interaction Network Metrics Tell Us About Specialization and Biological Traits.” Ecology 89: 3387–3399. 10.1890/07-2121.1. 19137945

[ece374021-bib-0007] Borgatti, S. P. 2002. NetDraw: Graph Visualization Software. Analytic Technologies.

[ece374021-bib-0008] Bosco, L. , Y. Xu , P. Deshpande , and A. Lehikoinen . 2022. “Range Shifts of Overwintering Birds Depend on Habitat Type, Snow Conditions and Habitat Specialization.” Oecologia 199: 725–736. 10.1007/s00442-022-05209-5. 35767049 PMC9309152

[ece374021-bib-0009] Calatayud, J. , E. Andivia , A. Escudero , et al. 2019. “Positive Associations Among Rare Species and Their Persistence in Ecological Assemblages.” Nature Ecology & Evolution 4: 40–45. 10.1038/s41559-019-1053-5. 31844189

[ece374021-bib-0010] CaraDonna, P. J. , W. K. Petry , R. M. Brennan , et al. 2017. “Interaction Rewiring and the Rapid Turnover of Plant–Pollinator Networks.” Ecology Letters 20: 385–394. 10.1111/ele.12740. 28156041

[ece374021-bib-0011] Clavel, J. , R. Julliard , and V. Devictor . 2011. “Worldwide Decline of Specialist Species: Toward a Global Functional Homogenization?” Frontiers in Ecol & Environ 9: 222–228. 10.1890/080216.

[ece374021-bib-0012] Connell, J. H. 1980. “Diversity and the Coevolution of Competitors, or the Ghost of Competition Past.” Oikos 35: 131. 10.2307/3544421.

[ece374021-bib-0013] Cui, J. 2024. Nutritional Niche Studies of Sympatric Anatidae Species (D). Qufu Normal University.

[ece374021-bib-0014] Dhondt, A. A. 2012. Interspecific Competition in Birds, Oxford Avian Biology Series. Oxford University Press.

[ece374021-bib-0016] Dong, D. , Z. Fan , Z. Li , et al. 2013. “Interspecific Associations Between Parus Major and Other Bird Communities in Beijing Xishan Region.” Acta Ecologica Sinica 33: 6614–6633. 10.5846/stxb201302060247.

[ece374021-bib-0017] Dupont, Y. L. , B. Padrón , J. M. Olesen , and T. Petanidou . 2009. “Spatio‐Temporal Variation in the Structure of Pollination Networks.” Oikos 118: 1261–1269. 10.1111/j.1600-0706.2009.17594.x.

[ece374021-bib-0018] Elmberg, J. , H. Pöysä , K. Sjöberg , and P. Nummi . 1997. “Interspecific Interactions and Co‐Existence in Dabbling Ducks: Observations and an Experiment.” Oecologia 111: 129–136. 10.1007/s004420050216. 28307498

[ece374021-bib-0020] Fortuna, M. A. , and J. Bascompte . 2006. “Habitat Loss and the Structure of Plant–Animal Mutualistic Networks.” Ecology Letters 9: 281–286. 10.1111/j.1461-0248.2005.00868.x. 16958893

[ece374021-bib-0100] Fortuna, M. A. , D. B. Stouffer , J. M. Olesen , et al. 2010. “Nestedness Versus Modularity in Ecological Networks: Two Sides of the Same Coin?” Journal of Animal Ecology 79: 811–817. 10.1111/j.1365-2656.2010.01688.x. 20374411

[ece374021-bib-0079] Fox, A. D. , and J. Madsen . 2017. “Threatened Species to Super‐Abundance: The Unexpected International Implications of Successful Goose Conservation.” Ambio 46: 179–187. 10.1007/s13280-016-0878-2. 28215012 PMC5316321

[ece374021-bib-0021] Fretwell, S. D. , and H. L. Lucas . 1969. “On Territorial Behavior and Other Factors Influencing Habitat Distribution in Birds: I. Theoretical Development.” Acta Biotheoretica 19: 16–36. 10.1007/BF01601953.

[ece374021-bib-0022] Gao, X. , Z. Guo , M. Zhang , X. Liang , M. Zhao , and L. Qin . 2024. “Ecological Source Identification and Ecological Security Pattern Construction From the Perspective of Ecosystem Service Supply and Demand: A Case Study of Baiyangdian Basin in China.” Environmental Development and Sustainability 27: 22947–22970. 10.1007/s10668-024-05302-0.

[ece374021-bib-0023] Gu, D. , Q. Zhao , and L. Cao . 2025. “Historical Changes in the Waterbird Community of Baiyangdian.” National Park 3: 377–383. 10.20152/j.np.202309091709.

[ece374021-bib-0024] Hamza, F. , A. M. Abdi , M.‐A. Chokri , et al. 2024. “Landscape Context and Wetland Attributes Influence Wintering Waterbirds in Important Bird and Biodiversity Areas: Implications for Conservation and Management.” Landscape Ecology 39: 151. 10.1007/s10980-024-01942-9.

[ece374021-bib-0080] HilleRisLambers, J. , P. B. Adler , W. S. Harpole , J. M. Levine , and M. M. Mayfield . 2012. “Rethinking Community Assembly Through the Lens of Coexistence Theory.” Annual Review of Ecology, Evolution, and Systematics 43: 227–248. 10.1146/annurev-ecolsys-110411-160411.

[ece374021-bib-0026] Huang, X. , B. Meng , H. Li , et al. 2024. “Interspecific Associations Between *Rhinopithecus brelichi* and Its Sympatric Species Using Infrared Cameras.” Biodiversity Science 32: 23402. 10.17520/biods.2023402.

[ece374021-bib-0027] Irigoin‐Lovera, C. , D. M. Luna , D. A. Acosta , and C. B. Zavalaga . 2019. “Response of Colonial Peruvian Guano Birds to Flying UAVs: Effects and Feasibility for Implementing New Population Monitoring Methods.” PeerJ 7: e8129. 10.7717/peerj.8129. 31844569 PMC6911346

[ece374021-bib-0028] IUCN . 2025. The IUCN Red List of Threatened Species. IUCN.

[ece374021-bib-0029] Jiang, N. , S. Wu , Z. Wang , and X. Yu . 2021. “Distribution Status of the Baer's Pochard in Shaanxi Province.” Chinese Journal of Zoology 56: 787–790. 10.13859/j.cjz.202105016.

[ece374021-bib-0081] Kraft, N. J. B. , P. B. Adler , O. Godoy , E. C. James , S. Fuller , and J. M. Levine . 2015. “Community Assembly, Coexistence and the Environmental Filtering Metaphor.” Functional Ecology 29: 592–599. 10.1111/1365-2435.12345.

[ece374021-bib-0032] Krause, J. , and G. D. Ruxton . 2002. Living in Groups. University PressOxford. 10.1093/oso/9780198508175.001.0001.

[ece374021-bib-0033] Lehikoinen, A. , Å. Lindström , A. Santangeli , et al. 2021. “Wintering Bird Communities Are Tracking Climate Change Faster Than Breeding Communities.” Journal of Animal Ecology 90: 1085–1095. 10.1111/1365-2656.13433. 33496011

[ece374021-bib-0035] Li, L. , F. Ma , J. Li , S. Geng , and C. Ding . 2024. “Habitat Use and Time‐Activity Budget of Wintering *Aythya baeri* in Minquan, Henan.” Chinese Journal of Zoology 59: 1–10. 10.13859/j.cjz.202421119.

[ece374021-bib-0036] Li, Q. , Z. Li , and Z. Liu . 2024. “Spatial Association Networks Reveal the Biological Communities of the Tibetan Macaque ( *Macaca thibetana* ) in Sichuan, China.” International Journal of Primatology 45: 774–809. 10.1007/s10764-024-00417-7.

[ece374021-bib-0037] Li, Q. , Y. Zhong , H. Zhou , et al. 2022. “Analysis on Interspecific Association of Sambars Community in Wolong National Nature Reserve, Sichuan, China.” Chinese Journal of Wildlife 43: 314–322. 10.12375/ysdwxb.20220203.

[ece374021-bib-0038] Li, S. , W. J. McShea , D. Wang , et al. 2020. “Retreat of Large Carnivores Across the Giant Panda Distribution Range.” Nature Ecology & Evolution 4: 1327–1331. 10.1038/s41559-020-1260-0. 32747773

[ece374021-bib-0040] Liang, D. , X. Pan , X. Luo , et al. 2021. “Seasonal Variation in Community Composition and Distributional Ranges of Birds Along a Subtropical Elevation Gradient in China.” Diversity and Distributions 27: 2527–2541. 10.1111/ddi.13420.

[ece374021-bib-0041] Liang, X. , X. Gao , M. Zhao , Z. Guo , and W. Shen . 2023. “Ecological Space Resilience Assessment of Baiyangdian Basin From the Perspective of Evolutionary Resilience.” Frontiers in Ecology and Evolution 11: 1200218. 10.3389/fevo.2023.1200218.

[ece374021-bib-0042] Liu, W. , C. Hu , J. Yin , B. Han , D. Yu , and H. Xu . 2020. “Status and Distribution of Potential Suitable Habitats of Bear's Pochard Population.” Wetland Science 18: 387–396. 10.13248/j.cnki.wetlandsci.2020.04.002.

[ece374021-bib-0043] Liu, Z. , Y. Zhong , X. Wang , et al. 2022. “Analysis on the Community Environment of Sichuan Snub‐Nosed Monkeys ( *Rhinopithecus roxellana* ) in Wolong, China.” Chinese Journal of Wildlife 43: 614–622.

[ece374021-bib-0044] Martín González, A. M. , B. Dalsgaard , and J. M. Olesen . 2010. “Centrality Measures and the Importance of Generalist Species in Pollination Networks.” Ecological Complexity 7: 36–43. 10.1016/j.ecocom.2009.03.008.

[ece374021-bib-0045] Mccarthy, J. M. , C. L. Hein , J. D. Olden , and M. Jake Vander Zanden . 2006. “Coupling Long‐Term Studies With Meta‐Analysis to Investigate Impacts of Non‐Native Crayfish on Zoobenthic Communities.” Freshwater Biology 51: 224–235. 10.1111/j.1365-2427.2005.01485.x.

[ece374021-bib-0046] Olesen, J. M. , J. Bascompte , Y. L. Dupont , and P. Jordano . 2007. “The Modularity of Pollination Networks.” Proceedings of the National Academy of Sciences of the United States of America 104: 19891–19896. 10.1073/pnas.0706375104. 18056808 PMC2148393

[ece374021-bib-0047] Pianka, E. R. 1974. “Niche Overlap and Diffuse Competition.” Proceedings of the National Academy of Sciences of the United States of America 71: 2141–2145. 10.1073/pnas.71.5.2141. 4525324 PMC388403

[ece374021-bib-0048] Post, E. , and M. C. Forchhammer . 2002. “Synchronization of Animal Population Dynamics by Large‐Scale Climate.” Nature 420: 168–171. 10.1038/nature01064. 12432390

[ece374021-bib-0049] Power, M. E. , D. Tilman , J. A. Estes , et al. 1996. “Challenges in the Quest for Keystones.” Bioscience 46: 609–620. 10.2307/1312990.

[ece374021-bib-0082] Pöysä, H. 1983. “Resource Utilization Pattern and Guild Structure in a Waterfowl Community.” Oikos 40: 295–307. 10.2307/3544595.

[ece374021-bib-0050] Prins, H. H. T. , and H. P. Van Der Jeugd . 1993. “Herbivore Population Crashes and Woodland Structure in East Africa.” Journal of Ecology 81: 305. 10.2307/2261500.

[ece374021-bib-0052] R Core Team . 2025. R: A Language and Environment for Statistical Computing. R Foundation for Statistical Computing.

[ece374021-bib-0053] Revelle, W. 2025. Psych: Procedures for Psychological, Psychometric, and Personality Research. Northwestern University.

[ece374021-bib-0054] Schluter, D. 1984. “A Variance Test for Detecting Species Associations, With Some Example Applications.” Ecology 65: 998–1005. 10.2307/1938071.

[ece374021-bib-0056] Shi, Y. 2015. The Research of Coexistence Pattern of Corvidae Birdsin Tiantong, Zhejiang Province (D). East China Normal University.

[ece374021-bib-0057] Siegel, S. , and N. J. Castellan Jr . 1988. Nonparametric Statistics for the Behavioral Sciences. 2nd ed. Mcgraw‐Hill Book Company.

[ece374021-bib-0058] Tian, Z. , D. Huo , K. Yi , J. Que , Z. Lu , and J. Hou . 2024. “Evaluation of Suitable Habitats for Birds Based on MaxEnt and Google Earth Engine—A Case Study of Baer's Pochard ( *Aythya baeri* ) in Baiyangdian, China.” Remote Sensing 16: 64. 10.3390/rs16010064.

[ece374021-bib-0059] Trøjelsgaard, K. , and J. M. Olesen . 2016. “Ecological Networks in Motion: Micro‐ and Macroscopic Variability Across Scales.” Functional Ecology 30: 1926–1935. 10.1111/1365-2435.12710.

[ece374021-bib-0060] Tylianakis, J. M. , E. Laliberté , A. Nielsen , and J. Bascompte . 2010. “Conservation of Species Interaction Networks.” Biological Conservation 143: 2270–2279. 10.1016/j.biocon.2009.12.004.

[ece374021-bib-0061] Vázquez, D. P. , N. P. Chacoff , and L. Cagnolo . 2009. “Evaluating Multiple Determinants of the Structure of Plant–Animal Mutualistic Networks.” Ecology 90: 2039–2046. 10.1890/08-1837.1. 19739366

[ece374021-bib-0062] Wang, C. , J. Que , Z. Zhang , D. Huo , and J. Hou . 2023. “Diversity of Bird Species in Winter in Baiyangdian Lake.” Journal of Hebei University (Natural Science Edition) 43: 298–302. 10.3969/j.issn.10001565.2023.03.010.

[ece374021-bib-0064] Wang, X. , M. Barter , L. Cao , J. Lei , and A. D. Fox . 2012. “Serious Contractions in Wintering Distribution and Decline in Abundance of Baer's Pochard *Aythya baeri* .” Bird Conservation International 22: 121–127. 10.1017/S0959270912000214.

[ece374021-bib-0065] Wang, X. , L. Shen , Y. Zhong , Z. Li , Z. Liu , and Q. Li . 2023. “Species Associations and Conservation of Sichuan Snub‐Nosed Monkey in the Tangjiahe National Nature Reserve.” Chinese Journal of Wildlife 44: 1–13. 10.12375/ysdwxb.20230101.

[ece374021-bib-0066] Wei, S. , H. JIa , Y. Chen , et al. 2020. “Reproductive Ecology of Baer's Pochard *Aythya baeri* in South China.” Wild 70: 211–227.

[ece374021-bib-0067] Wetlands International . 2018. Guidance on Waterbird Monitoring Methodology: Field Protocol for Waterbird Counting. Wetlands International.

[ece374021-bib-0068] Wickham, H. 2016. ggplot2: Elegant Graphics for Data Analysis. Springer‐Verlag.

[ece374021-bib-0069] Wickham, H. , R. François , L. Henry , K. Müller , and D. Vaughan . 2023. “dplyr: A Grammar of Data Manipulation.” https://dplyr.tidyverse.org.

[ece374021-bib-0070] Wiegand, T. , S. Gunatilleke , and N. Gunatilleke . 2007. “Species Associations in a Heterogeneous Sri Lankan Dipterocarp Forest.” American Naturalist 170: E77–E95. 10.1086/521240. 17891727

[ece374021-bib-0071] Wisz, M. S. , J. Pottier , W. D. Kissling , et al. 2013. “The Role of Biotic Interactions in Shaping Distributions and Realised Assemblages of Species: Implications for Species Distribution Modelling.” Biological Reviews 88: 15–30. 10.1111/j.1469-185X.2012.00235.x. 22686347 PMC3561684

[ece374021-bib-0072] Wu, P. , X. Peng , S. Yang , et al. 2019. “Spatial Distribution Patterns and Correlation of *Tamarix chinensis* Population in Coastal Wetlands of Shandong, China.” Chinese Journal of Plant Ecology 43: 817–824.

[ece374021-bib-0073] Yang, H. , J. Li , N. Jiang , X. Shi , P. Wang , and Z. Li . 2021. “Interspecific Associations Between Takins ( *Budorcas taxicolor* ) and Sympatric Terrestrial Mammals at Wolong National Nature Reserve, China.” Chinese Journal of Wildlife 42: 654–662. 10.19711/j.cnki.issn2310-1490.20210531.001.

[ece374021-bib-0074] Yang, W. , J. Yan , Y. Wang , B. Zhang , and H. Wang . 2020. “Seasonal Variation of Aquatic Macrophytes and Its Relationship With Environmental Factors in Baiyangdian Lake, China.” Science of the Total Environment 708: 135112. 10.1016/j.scitotenv.2019.135112. 31787312

[ece374021-bib-0075] Yuan, S. , and X. Wang . 2023. “Niche and Interspecific Association of Dominant Tree Species in Spruce–Fir Mixed Forests in Northeast China.” Forests 14: 1513. 10.3390/f14081513.

[ece374021-bib-0076] Zhang, J. 2016. “spaa: SPecies Association Analysis.” https://github.com/helixcn/spaa.

[ece374021-bib-0077] Zheng, Z. , X. Chen , X. Luo , and F. Zhang . 2017. “Investigation and Analysis of Seed Plants Resources in Baiyangdian Wetland.” Journal of Hebei University (Natural Science Edition) 37: 440–448. 10.3969/j.issn.1000-1565.2017.04.017.

[ece374021-bib-0078] Zhu, Y. , F. Bai , H. Liu , et al. 2011. “Population Distribution Patterns and Interspecific Spatial Associations in Warm Temperate Secondary Forests, Beijing: Population Distribution Patterns and Interspecific Spatial Associations in Warm Temperate Secondary Forests, Beijing.” Biodiversity Science 19: 252–259. 10.3724/SP.J.1003.2011.08024.

