# Supplementary material for: Seasonal Role Reversal of Baer's Pochard in Waterbird Association Networks
Source: Ecol Evol. 2026 Aug 4;16(8):e74021. doi: 10.1002/ece3.74021 (PMC13437952; doi:10.1002/ece3.74021)
Supplement: Supplementary file 1 [file ECE3-16-e74021-s005.docx]

**Appendix Table 1. Checklist of waterbird species sympatric with Baer's Pochard in Baiyangdian Wetland**

| **Number** | **Species** | **Residency type** | **Recording period** | **Number of individuals** |
| --- | --- | --- | --- | --- |
|  | **Anseriformes** |  |  |  |
|  | **Anatidae** |  |  |  |
| 1 | *Cygnus columbianus* | W | W | 26 |
| 2 | *Anser cygnoides* | P | B | 8 |
| 3 | *Bucephala clangula* | W | W | 4 |
| 4 | *Mergellus albellus* | W | A | 31 |
| 5 | *Mergus merganser* | W | W | 55 |
| 6 | *Aix galericulata* | P | B | 4 |
| 7 | *Netta rufina* | R | A | 97 |
| 8 | *Aythya ferina* | S | A | 80 |
| 9 | *Aythya baeri* | R | A | 619 |
| 10 | *Aythya nyroca* | R | A | 1174 |
| 11 | *Aythya fuligula* | P | A | 56 |
| 12 | *Spatula querquedula* | W | A | 3 |
| 13 | *Spatula clypeata* | W | A | 16 |
| 14 | *Sibirionetta formosa* | W | W | 4 |
| 15 | *Mareca falcata* | P | A | 849 |
| 16 | *Mareca strepera* | R | A | 882 |
| 17 | *Mareca penelope* | W | A | 6 |
| 18 | *Anas zonorhyncha* | R | A | 1776 |
| 19 | *Anas platyrhynchos* | R | A | 2139 |
| 20 | *Anas acuta* | R | A | 10 |
| 21 | *Anas crecca* | P | A | 126 |
|  | **Podicipediformes** |  |  |  |
|  | **Podicedidae** |  |  |  |
| 22 | *Tachybaptus ruficollis* | R | A | 200 |
| 23 | *Podiceps cristatus* | R | A | 375 |
|  | **Gruiformes** |  |  |  |
|  | **Rallidae** |  |  |  |
| 24 | *Gallinula chloropus* | R | A | 149 |
| 25 | *Fulica atra* | R | A | 27388 |
|  | **Pelecaniformes** |  |  |  |
|  | **Threskiorothidae** |  |  |  |
| 26 | *Platalea leucorodia* | P | B | 31 |
|  | **Ardeidae** |  |  |  |
| 27 | *Botaurus stellaris* | P | W | 1 |
| 28 | *Ixobrychus sinensis* | S | B | 21 |
| 29 | *Nycticorax nycticorax* | S | B | 36 |
| 30 | *Ardeola bacchus* | S | B | 28 |
| 31 | *Ardea cinerea* | S | A | 45 |
| 32 | *Ardea purpurea* | S | B | 57 |
| 33 | *Ardea alba* | S | B | 3 |
| 34 | *Ardea intermedia* | S | B | 1 |
| 35 | *Egretta garzetta* | R | A | 120 |
|  | **Suliformes** |  |  |  |
|  | **Sulidae** |  |  |  |
| 36 | *Phalacrocorax carbo* | P | A | 3 |
|  | **Charadriiformes** |  |  |  |
|  | **Recurvirostridae** |  |  |  |
| 37 | *Himantopus himantopus* | S | B | 51 |
|  | **Charadriidae** |  |  |  |
| 38 | *Vanellus cinereus* | P | B | 3 |
| 39 | *Charadrius dubius* | S | B | 15 |
|  | **Scolopacidae** |  |  |  |
| 40 | *Gallinago gallinago* | P | B | 43 |
| 41 | *Actitis hypoleucos* | S | B | 1 |
| 42 | *Tringa ochropus* | P | B | 6 |
| 43 | *Tringa erythropus* | P | B | 35 |
| 44 | *Tringa nebularia* | P | B | 67 |
| 45 | *Tringa glareola* | P | B | 7 |
|  | **Laridae** |  |  |  |
| 46 | *Chroicocephalus ridibundus* | W | B | 31 |
| 47 | *Chlidonias hybrida* | S | B | 634 |
| 48 | *Chlidonias leucopterus* | S | B | 5 |

Note: For residency type: S = summer visitor; R = resident; W = winter visitor; P = passage migrant. For recording period: B = recorded during the breeding season; W = recorded during the wintering season; A = recorded during both seasons. Number of individuals refers to the cumulative count recorded across both seasons.
